# The effects of long-term total parenteral nutrition on gut mucosal immunity in children with short bowel syndrome: a systematic review

**DOI:** 10.1186/1472-6955-4-2

**Published:** 2005-02-01

**Authors:** Beyhan Duran

**Affiliations:** 1 School of Nursing, University of Connecticut, Storrs, Connecticut, USA; 2 Children's Clinical Research Center, Yale-New Haven Children's Hospital, New Haven, Connecticut, USA; 3 (Time of Writing) Infant-Toddler/Pediatric Respiratory Care Unit, Yale-New Haven Children's Hospital, New Haven, Connecticut, USA

## Abstract

**Background:**

Short bowel syndrome (SBS) is defined as the malabsorptive state that often follows massive resection of the small intestine. Most cases originate in the newborn period and result from congenital anomalies. It is associated with a high morbidity, is potentially lethal and often requires months, sometimes years, in the hospital and home on total parenteral nutrition (TPN). Long-term survival without parenteral nutrition depends upon establishing enteral nutrition and the process of intestinal adaptation through which the remaining small bowel gradually increases its absorptive capacity. The purpose of this article is to perform a descriptive systematic review of the published articles on the effects of TPN on the intestinal immune system investigating whether long-term TPN induces bacterial translocation, decreases secretory immunoglobulin A (S-IgA), impairs intestinal immunity, and changes mucosal architecture in children with SBS.

**Methods:**

The databases of OVID, such as MEDLINE and CINAHL, Cochran Library, and Evidence-Based Medicine were searched for articles published from 1990 to 2001. Search terms were total parenteral nutrition, children, bacterial translocation, small bowel syndrome, short gut syndrome, intestinal immunity, gut permeability, sepsis, hyperglycemia, immunonutrition, glutamine, enteral tube feeding, and systematic reviews. The goal was to include all clinical studies conducted in children directly addressing the effects of TPN on gut immunity.

**Results:**

A total of 13 studies were identified. These 13 studies included a total of 414 infants and children between the ages approximately 4 months to 17 years old, and 16 healthy adults as controls; and they varied in design and were conducted in several disciplines. The results were integrated into common themes. Five themes were identified: 1) sepsis, 2) impaired immune functions: *In vitro *studies, 3) mortality, 4) villous atrophy, 5) duration of dependency on TPN after bowel resection.

**Conclusion:**

Based on this exhaustive literature review, there is no direct evidence suggesting that TPN promotes bacterial overgrowth, impairs neutrophil functions, inhibits blood's bactericidal effect, causes villous atrophy, or causes to death in human model.

The hypothesis relating negative effects of TPN on gut immunity remains attractive, but unproven. Enteral nutrition is cheaper, but no safer than TPN. Based on the current evidence, TPN seems to be safe and a life saving solution.

## Background

In the late 1960's, the introduction of total parenteral nutrition (TPN) as an alternative nutrition provided a life saving solution to children with chronic bowel obstructions, fistulas, loss of mucosal body surfaces, short bowel syndrome, and other clinical problems that precluded enteral diet by mouth or tube feeding for long periods of time. Intravenous administration of TPN became an essential fluid to meet nutritional needs and to avoid progressive starvation-induced malnutrition, which changed the outcome of patients from dying [1]. Since then, TPN has been a gold standard practice in treatment and a panacea for infants and children who are unable to eat or to absorb enterally provided nutrients [1-4]. As a result, the prognosis for patients with SBS has changed dramatically and the management with the expected survival for infants with congenital gastrointestinal anomalies and gut failure have improved significantly [5,6].

However, its use has been shown to associate with an increased incidence of infection [7]. A number of independent experimental studies have been carried out shown that intravenous TPN negatively influences gut barrier functions and mucosal immunity while withholding nutrients by mouth or enteral tube feeding, after the resection of small intestine. These studies demonstrated that TPN is associated with: 1) increases in intestinal permeability, bacterial overgrowth, and bacterial translocation, 2) rapid changes in gut-associated lymphoid tissue (GALT) T cells, B cell, and secretory immunoglobulin A (S-IgA) levels, 3) impairment in IgA-mediated mucosal immunity defenses in the respiratory tract, 4) impairment in neutrophil function, 5) alteration in gastrointestinal (GI) architecture or mucosal atrophy [8-14].

This paper presents a descriptive systematic review of published research articles on the effects of the long-term TPN on gut mucosal immunity in children with SBS; specifically, it addresses whether TPN: 1) promotes bacterial translocation, 2) impairs intestinal mucosal immunity by decreasing S-IgA levels, 3) inhibits neutrophil and cytokine functions in blood, 4) promotes atrophy of the mucosal villi, 5) hyperglycemia, and 6) causes death. It is hoped that these findings will expand the knowledge of pediatric nurses, and have an impact on clinical practice by being included in the pediatric parenteral nutritional guidelines. Since the review of literature did not reveal any systematic reviews of TPN and mucosal immunity on children with SBS, the aim of this descriptive systematic review of individually published scientific studies is to impart a better understanding of the effects of TPN on gut mucosal defense and barrier function.

Short bowel syndrome, one of the major indications for diagnostic categories using long-term TPN [15], is a clinically complex disorder resulting from multiple alterations of normal intestinal anatomy and physiology and producing a variety of nutritional, infectious, and metabolic complications because of impairment caused by vascular disease, intestinal volvulus, ischemic bowel, inflammatory bowel, and necrotizing enterocolitis (NEC) [16,17]. SBS is also described as, " the malabsorptive state" that mostly follows massive resection of the small intestine [18,19].

After resection, the residual or remaining small bowel undergoes intestinal adaptation, a process characterized by mucosal hyperplasia, villus lengthening, increased crypt depth (intestinal gland), and bowel dilation. The process of adaptation is complex and includes both structural and functional changes. The earliest sign can be detected within 24-48 hours, and process may continue for months, possibly years. In the early phase, mucosal hyperplasia and villus hypertrophy occur. Oral nutrients and hormones stimulate this intestinal adaptation. The main clinical challenge in SBS lies in managing the many nutritional problems that occur as a result of malabsorption secondary to the reduced absorptive surface area [20,21].

### Anatomy and physiology of the gastrointestinal immune system

The normal length of intestine in full-term newborn is estimated at 200 cm-250 cm [22], and the normal adult small intestine is about 400 cm [23]. The small intestine extends from the pylorus to the ileocecal valve. It is functionally divided into three segments: the *duodenum*, the *jejunum*, and the *ileum*. The duodenum begins at the pylorus and ends where it joins the jejunum. The end of jejunum and beginning of the ileum are not distinguished by an anatomic marker[24]. The ileocecal valve functions as a barrier to prevent both reflux of colonic contents into small intestine and rapid passage of contents through the ileum [25].

The primary function of the small intestine is to absorb nutrients into the circulation [26]. During the course of this activity the intestine is exposed to a wide variety of antigens derived from foods, resident bacteria, and invading microorganisms. All products of carbohydrate, protein, and fat digestion, as well as most of the ingested electrolytes, vitamins, and water are normally absorbed by small intestine indiscriminately. Normally, nutrient digestion and absorption occur predominantly in the upper intestine. The jejunum has a larger absorptive surface due to longer villi, and a higher concentration of mucosal digestive enzymes and transport proteins, whereas the ileum has shorter villi. Very little absorption occurs in the ileum, not because the ileum does not have absorptive capacity but because most absorption has been already accomplished before the intestinal contents reach the ileum[21,25,27].

The tight junctions of the jejunum are highly porous, so that exposure of the mucosa to hyperosmolar nutrient solutions leads to efflux or flow of water and electrolytes into the lumen (interior space of intestine). This fluid is then reabsorbed in both the ileum and colon. The distal ileum has specialized transport carriers for the absorption of bile salts and vitamin B_12. _The ileocecal valve forms an important barrier, slowing transit from the small intestine to the colon and limiting bacterial colonization of the small intestine [21]. Ileocecal valve prevents retrograde colonization of distal small bowel to a significant extent. In the absence of the valve, however, free reflux of right-sided liquid colonic content occurs, and the total load of colonic bacteria exposed to the distal intestine increases greatly [28].

Histological organization of the intestinal wall is divided into four major layers. These major layers include: (1) the *mucosa*, the innermost layer, which comprises *epithelium*, *lamina propria*, and *muscularis *layers; (2) The *submucosa *consists largely of loose connective tissue with collagen and elastin fibrils. Glands, nerve trunks and blood vessels are also present in the submucosa; (3) The next layer, the *muscularis*, typically consists of two substantial layers of smooth muscle cells; an inner circular layer and an outer longitudinal layer; and (4) The *serosa *is the outermost layer of intestinal tract [25,29-31].

The mucosa is considered an "architectural marvel." The epithelial layer of mucosa regulates absorptive, secretory, and protective barrier functions. This thin epithelial layer composed of columnar absorptive cells, goblet cells, undifferentiated crypt epithelial cells, panet cells, enteroendocrine cells, tuft cells, cup cells, and intraepithelial lymphocytes [32]. This mucosal layer covers the villus, a finger like projection that is made up of epithelial cells called enterocytes and its crypt or submucosal gland, and is responsible for absorption of electrolytes, water, and nutrition. The enterocyte surface contains special luminal projections called microvilli, which provide an increased surface area that is referred to as brush border membrane. Although not part of the epithelium, mucus on the surface of the mucosa shields the mucosal epithelial cells from direct contact with the intestinal luminal environment [29,32,33].

Beneath the mucosal epithelium is the connective and supportive tissue called the lamina propria. The lamina propria contains various immunocompetent cells, including plasma cells, mast cells, macrophages, and lymphocytes that produce not only immunoglobulins but also cytokine mediators [32].

### Mucosal defense system

#### Nonimmune antibacterial factors

A complex intestinal mucosal defense system plays an important role to prevent invasion of gut bacteria or absorption of endotoxin. Peristaltic waves and contractions sweep the intestinal contents in a steady distal flow toward the colon, which is a major importance in reducing the growth of bacteria in the proximal intestine [28]. During fasting, motilin, a putative regulatory peptide, is believed to be one mechanism that initiates the cyclic motility pattern termed the migrating motor complex (MMC), which functions as a gastrointestinal "housekeeper" by sweeping lumenal content and bacteria from stomach and small bowel into colon[34].

Mucus, an epithelial secretion, also helps reducing bacterial overgrowth. Mucus has special properties that enable to trap bacteria in an intraluminal location while moving organisms distally in bolus fashion. Carbohydrate serves as a nutrient for some bacteria, thus attracting them to a mobile colonization site that is continuously replaced. Enzymatic digestion also significantly reduces bacterial overgrowth. Pancreatic juice has antibacterial activity. Intestinal secretions from the stomach, intestine, and pancreas are a significant deterrent to growth due to their ability to dilute the bacterial mass [28,32].

Gastric acidity acts as an initial line of defense against ingested bacteria. Gastric juice with pH less than 4.0 is bactericidal for most organisms, although not immediately [28]. In one *in vitro *study, bacteria instilled into an intact lumen of a normal human stomach were promptly killed in less than 15 minutes at a pH of less than 3.0, but remained viable in the achlorhydric stomach for at least 1 hour [35]. Chronic inhibition of gastric acid secretion by histamine_2 _(H_2_) receptor blockade in healthy adults, however, has been shown to increase the number of gastric bacteria [36].

The intestinal tight junctions between epithelial cells and permeability have been reviewed [37]. Permeability refers to the ability of small molecules to penetrate the gastrointestinal mucosa. The tight junctions are an important part of the intestinal barrier. Tight junctions are dynamic structures, and their barrier function may be modulated by nutrients such as glucose and amino acids as well as bacterial toxins and chemotaxins. Loss of intestinal barrier is associated with translocation of enteric bacteria in animal modal, but in humans bacterial translocation is not associated with increased intestinal permeability or villous atrophy. Glutamine, a nonessential amino acid, is considered to be the principal respiratory fuel for enterocytes. A lack of glutamine promotes mucosal atrophy and increases intestinal permeability [37-39].

Thomson and colleagues [40-42], citing various sources, suggest that enterocytes function as "nonclassical" immune cells, which play a major role as a source of proinflammatory cytokines and cytotoxins. A key proinflammatory mediator produced in intestinal mucosa is the free radical nitric oxide. Nitric oxide (NO), a pluripotent signaling and effector molecule, is increased with mild acidosis and enhances intestinal permeability. Nitric oxide is produced as a result of conversion of arginine to citrulline by the enzyme NO synthase (NOS). Nitric oxide has potent bactericidal effects against a wide variety of micro-organisms, including the majority of the intestinal microflora. Furthermore, arginine supplementation has been shown to improve survival in a guinea-pig model of peritonitis. Inhibition of NO synthesis has been shown to increase intestinal permeability via mast cells, which suggests that NO may regulate intestinal barrier function [43,44].

The permeability of the intestine can be increased by variety of factors, such as psychological stress, fasting, and certain drugs. During fasting or malnutrition, intestinal secretion of ions and fluid increased, permeability to ions and macromolecules is increased and associated mucosal atrophy reduces intestinal absorption of nutrients. Some drugs also alter intestinal permeability. Nonsteroidal anti-inflammatory drugs (NSAIDs) increase intestinal permeability in both animals and humans. Glutamine, the major source of the small intestinal mucosa, may be partially normalize this NSAID-induced permeability [37,45].

#### Immunological factors

The gastrointestinal track or gut associated lymphoid tissue (GALT) has an important role in gut immunity. The gut is considered the largest lymphoid organ in the body housing more than 10^12 ^lymhocytes and more antibody (secretory IgA) than any other site in the body including spleen [46]. The lamina propria plasma cells produce secretory immunoglobulin A (S-IgA) in response to food antigens and microorganisms. Secretory IgA coats the lumen or interior surface of small intestine and prevents any microbial pathogens or viruses penetrating the epithelial layer and passing into the other organs [29,47].

S-IgA contains antibodies directed to biologically active antigens such as viruses, bacteria, enterotoxins, and enzymes normally restricted to the intestinal tract. A reduction in S-IgA level results in a greater frequency of GI infections and impaired reticuloendothelial or macrophage function, which predispose the child to systemic bacteremia [12,46,48].

### The effects of surgery or trauma on mucosal immunity

After surgical intervention, sympathetic response is activated creating a positive relationship between the severity of injury and the plasma concentration of adrenalin, noradrenalin and dopamine [49]. Following injury, the inflammatory cytokines, such as tumor necrosis factor (TNF), interleukin-1 (IL-1) and interleukin-6 (IL-6) are also released, which act only locally [39]. Cytokines are hormonelike peptides or intracellular signaling proteins released from white blood cells and all nucleated cells. They function by serving as chemical messengers within the immune system, and also communicate with certain cells in the nervous system. They have immune modulating effects, in which they work in parallel with other signal arising from direct cell-to-cell contact, providing a communication network involved in everyday function of the immune response [50-52].

In the systemic level, counter-regulatory hormones, such as adrenocorticotropic hormone (ACTH), antidiuretic hormone (ADH), catecholamines and stress hormone, cortisol are released. These cytokines and systemic hormones working together cause the multiorgan system failure. They also induce hypercatabolism, which is characterized by protein breakdown within skeletal muscle, accelerated breakdown of branched-chain amino acids and increased release of glutamine and alanine into systemic amino-acid pool. Glutamine, conditionally essential amino acid, is critical as an energy source for enterocytes, immune cells, and rapidly growing tissues. Overall, physiological stress response results with increased intestinal permeability and bacterial translocation, which promotes sepsis or multiorgan failure and potantiate hypercatabolism and protein-calorie malnutrition [39].

During illness, stress increases the concentration of counterregulatory hormones (glucagons, epinephrine, cortisol, and growth hormone) and cytokines. Counterregulatory hormones increase serum blood sugars by increasing hepatic glucose production and by decreasing peripheral glucose uptake [53]. Glucose turnover is increased in sepsis and trauma, but glucose is oxidized with reduced efficiency. Fat becomes a preferred energy substrates in septic patients. In critical surgical illness the rates of both protein synthesis and catabolism are increased. However, the increase in catabolism is of greater magnitude resulting in a net breakdown of protein and, if prolonged, immune compromise [49]. Overall, this leads to prominent metabolic derangements composed of high release and low use of glucose, amino acids, and free fatty acids (FFA), resulting in increased blood levels of these substrates. Increased levels of glucose and FFA have stimulating effects on inflammatory signaling leading to additional release of proinflammatory mediators and endothelial and neutrophil dysfunction. Insulin has the inherent capability to counteract the metabolic changes in septic patients [54].

### Complications of SBS

The most important ongoing complications in children with SBS reported in the literature are: recurrent sepsis, catheter related sepsis, TPN-associated cholestasis, metabolic disturbances, hyperglycemia, electrolyte imbalances, hypertriglyceridemia, gastric hypersecretion, diarrhea, and organ dysfunction [15,55-57].

#### Bacterial overgrowth

Bacterial overgrowth is defined as the presence of potentially pathogenic microorganism (PPM) in high concentration (≥ 10^5 ^colony forming units/mL) [58]; that is, increased numbers and species of bacteria in the small intestine. Bacterial growth in the normal bowel is controlled by gastric acid, pancreatic enzyme activity, enterocyte turnover, normal peristaltic activity in the small intestine, and ileocecal valve [59]. Bacterial overgrowth is found in children who have no ileacecal valve, the primary means for preventing reflux of bacteria from the colon into the small intestine. Progressive dilation of the small intestine as part of the adaptation response limits the efficacy of peristalsis in ridding the small intestine of bacteria. The diagnosis of bacterial overgrowth is made by culture of jejunal aspirate or by breath hydrogen testing.

Bacterial translocation is a phenomenon where intestinal pathogenic microorganisms, which are normally resident within the lumen of the intestinal tract, travel from the gut lumen into local mesenteric lymph nodes, and from there to distant sites, such as upper gastrointestinal tract, thereby causing sepsis and septicemia [8,60-62]. *Sepsis *in infants and children is defined as the systemic response to a possible infection. Evidence of bacteremia or an infectious focus is not required. The term *septicemia *is used when organisms or their toxic products are identified in the bloodstream, in other words, a pathogen is recovered from blood cultures or identified [63].

Most studies of the pathogenesis of sepsis in animals have been carried out shown that gut mucosa is particularly susceptible to injury through a variety of mechanisms, such as decreased mucosal blood flow, increased oxygen demand, decreased oxygen delivery, and reperfusion injury [64]. Sepsis is also associated with metabolic and inflammatory response to trauma or surgical illness. It has been suggested that the neuroendocrine response to sepsis and trauma results in metabolic changes; and inflammatory mediators released from wound itself or from a septic focus may play a part in these changes.

It is also widely believed that these infectious complications are related to the central venous access devices because the most recurring infection was reported as line infection [8,58,60,65,66]. However, it has been suggested that lipid emulsions of TPN may impair host defense, and in particular the bactericidal and migratory functions of the neutrophil polymorhonuclear (PMN) granulocyte in infants receiving TPN [7,51,60,67-69]. A large case-control study [70] involving 882 infants in two neonatal intensive care units, and one experimental animal study [71] conducted suggest that administration of lipids in parenteral nutrition regimens may cause phagocyte dysfunction, resulting in infectious complications such as bacteremia, pneumonia, and wound abscesses. In addition, these studies also suggest that the lipid component of TPN, which constitutes only long-chain triglycerides, possesses immunosuppressive properties that interfere with binding of interleukin 2 (IL-2), a product of activated T (helper) cell or cytokine, to its cellular receptor and impairs host defense, in particular the bactericidal and migratory functions of the neutrophil polymorphonuclear (PMN) granulocyte [50,69-72]. However, three randomized control clinical trials [73-75], and a case study with 4 patients [76] did not show that intralipid in TPN is associated with any immunosuppression. Interestingly, in contrast to others, one of the randomized control trials [73] showed that, rather than being immunosuppressive, lipid emulsions of TPN had immunostimulatory properties.

#### D-lactate acidosis

Bacterial overgrowth frequently complicates, especially when the ileocecal valve is absent and the dysmotility is present in the remaining bowel loops. In the colon, unabsorbed carbohydrates undergo bacterial fermentation to D-lactate, short-chain fatty acids (acetate, propionate and butyrate), which can not be metabolized by D-lactate dehydrogenase; and these small molecules are absorbed from colon, "carbohydrate salvage" [21,77]. Absorption of this metabolite (D-lactate) may cause neurological symptoms, metabolic acidosis with increased anion gap in these patients [55]. It is called, "*D-lactic acidosis Syndrome." *The episode of this syndrome causes patients develop slurred speech, ataxia, and altered affect. These patients appear "drunk." This condition is resolved after placing patient on a diet with restricted carbohydrate intake and metronidazole [23].

## Methods

In this review, a descriptive systematic, non-quantitative literature review was conducted using methodological guidelines [78-80]. A systematic review is a technique that uses systematically guided strategies to locate, select and critically appraise relevant original studies for its subjects, and summarizes the results that address a specific clinical question from studies that are included in the review. It is either descriptive (non-quantitative) when results of primary studies are summarized, but not statistically combined, or quantitative, in which statistical methods are used to combine results, also known as meta-analysis [80-83].

A descriptive systematic review is also called a *synthesis of literature*. This form of literature synthesis involves tabulation of study characteristics and results to summarize their findings in an area, and it also leads to new conclusions and knowledge as a result of systematically pulling together the fragmented results from single studies[80,84].

### Selecting and appraising studies for systematic review

To select and assess the methodological quality of the research studies to be included in this review, the author used a list of standardized criteria or the guidelines (Table 1) proposed by a group of researchers [78,82,85-89]. Studies were excluded if their published method section was not clear or absent; TPN was not used; study outcomes were not mucosal immunity related; or participants were not children.

**Table 1:** Study selection criteria

Review Question:	
	
To what extent does long-term TPN affect the intestinal immune system of infants who undergo bowel resection with no enteral nutritional support?	
Population	Were study patients pediatric age groups between newborn to 17 years old? Did study patients have intestinal resection prior to TPN given? Did study patients have documented bacterial infection after TPN started? Did study patients have documented impaired mucosal immunity after TPN started?
Study Intervention	Did at least one study group received Intravenous (IV-TPN? Did study group received IV-TPN more than10 days?
Control Intervention	Did one study group receive enteral feeding, but no IV-TPN?
Outcomes	Was one of the measured outcomes documented as bacterial translocation, villi atrophy, impaired immuno-function, and death?

Note. Modified Table from "Selecting and appraising studies for systematic review," by M. O. Meade and

W. S. Richardson, 1997, *Annals of internal medicine*, 127 (7), p. 531-537 [86]. Copyright 2002 by the American College of Physicians-American Society of Internal Medicine. Adapted with permission.

### Research question

To what extent does long-term TPN affect the intestinal immune system of infants who undergo bowel resection with no enteral nutritional support?

### Search strategies

For the purpose of this paper, searches of the following resources were used to do a comprehensive and exhaustive literature search: 1) Electronic bibliographic databases, 2) Reference lists from relevant primary and review articles, 3) The Internet, 4) Hand-searched journals, and 5) Unpublished studies, such as Thesis and Dissertation Abstracts [80].

A literature search was conducted using the following online databases: OVID Databases (MEDLINE, BIOSIS, CINAHL, *Current Contents*, HealthStar, OVID Full text journal articles), Cochran Library, Dissertation Abstracts, Thesis, PubMed, Web of Science, Evidence-Based Medicine, Evidence-Based Nursing, Journal Citation Reports, MD Consult, Academic Search, CancerLit, and Journal Collection Databases (Academic/IDEAL, Blackwell Science, Elsevier, and SpringerLink).

The newer articles provided a reference list of citations of previous articles. The research covered the years from 1990 through 2001 for original articles published in English. The following subject heading terms were included: Total parenteral nutrition, bacterial translocation, immunity, infant, children, short bowel-syndrome, short gut syndrome, intestinal immunity, epithelial permeability, immunonutrition, hyperglycemia, and sepsis. To prevent this systematic review from publication bias, unpublished studies were searched via Dissertation Abstract Online (FirstSearch) database, and one unpublished thesis relevant to this review was included [90].

### Sample

In this systematic review, 12 published studies using quantitative methods met the clinical trials criteria. In addition, one unpublished study was included in this review. These 13 studies included a total of 414 infants and children between the ages approximately 4 months to 17 years old, and 16 healthy adults as controls. One of the 13 clinical trial samples was not counted because the same sample was used for secondary analysis [58,60].

Although high quality, well-designed study, or the preferred research design for a review would be the one that randomly allocates (concealing the assignment code) the participants with the condition of interest to alternative therapeutic interventions, but when prospective randomized clinical trials (PRCT) are not possible to find or not available, NHS Center for Review and Dissemination Guidelines suggest that the next best available evidence should be considered based on the hierarchy of study designs, which are from highest to lowest level: 1) prospective experimental studies (e.g. randomized control trial with concealed allocation), 2) experimental study without randomization (quasi-experimental), 3) observational study with control group, a) cohort, b) case-control studies, 4) observational studies without control group, a) cross-sectional, b) before-and-after study, c) case series, and 5) expert opinion [80,91,92]. In this review, the following available research designs are included: two prospective experimental-control clinical trials [90,93], one case-control [94], two cohort studies [58,60], three *in-vitro *studies [7,51,67], one case study [95], and four retrospective medical chart reviews [5,96-98]. The studies were published between 1993 and 2001 in pediatric journals, annals of surgery, nutritional science journals, and immunology.

The descriptive methodological and characteristics of the samples are given in Table 2.

**Table 2:** Methodological Characteristics of the Clinical Trials Included in This Systematic Review

Studies	Discipline	Year	Publication	Country	Funding	Trial Type (Setting)	Participants
Andorsky et al. [96]	Surgery	2001	J. Pediatrics	USA	Yes	Retrospective (Hospital)	Neonates
Okada, Klein, Pierro, et al. [20]	Pediatric Surgery	1999	J Pediatric Surgery	UK	Yes	In Vitro, (Hospital)	Infants and adults
Okada, Klein, van Saene, et al. [7]	Pediatrics	2000	Annals of Surgery	UK	Yes	In Vitro, (Hospital)	Infants
Okada, Papp, et al. [19]	Pediatric Surgery, Immunobiology	1999	J Pediatric Surgery	UK	Yes	In Vitro (Hospital)	Infants and adults
Bines et al. [95]	Gastroenterology & Clinical Nutrition	1998	J. Pediatric Gastroenterology and Nutrition	Australia	Yes	Case study (Hospital)	Infants and children
Sondheimer et al [97]	Pediatrics	1998	J. Pediatrics	USA	No	Retrospective (Hospital)	Neonates
Kaufman et al. [98]	Pediatric Gastroenterology & Ped. Surgery	1997	J. Pediatrics	USA	Yes	Retrospective (Hospital)	Infants and children
Pierro, van Saene, Donnel, et al. [17]	Pediatric Surgery	1996	Archives of Surgery	UK	No	Cohort study (Hospital)	Infants
Pierro, van Saene, Jones, et al [18]	Pediatric Surgery	1998	Annals of Surgery	UK	No	Cohort study (Hospital)	Infants
Weber [94]	Pediatric Surgery	1995	J Pediatric Surgery	USA	No	Case-control (Hospital)	Infants
Chaet et al. [5]	Pediatric Surgery	1994	J Pediatric Gastroenterology & Nutrition	USA	No	Retrospective (Hospital)	Children
Rossi et al. [93]	Pediatric Gastroenterology	1993	Digestive Disease and Sciences	USA	No	Experimental-Control (Hospital)	Infants and children
Dahlstrom [90]	Pediatrics	1988	Unpublished Thesis	Sweden	Yes	Experimental-control (Hospital)	Infants and children

### Data analysis

To collate and present the extracted data, a coding sheet was used to collect information or study characteristics that included both demographic and methodological from the retrieved primary studies [79,99]. The results of the retrieved primary studies were reviewed and summarized in a coherent manner by using the NHS Center for Reviews and Dissemination [80] non-quantitative data synthesis guidelines, and classifying and structuring processes [100]. The data synthesis processes involve categorizing and classifying extracted data results, tabulation of each characteristic of the study and the major findings related to TPN, and then drawing a conclusion or making a coherent integrated report from the individual studies. Once the results were extracted, they were organized, and then categorized into similar themes. For example, the results related to sepsis, septicemia, bacterial overgrowth and bacterial translocation are grouped under sepsis. Five major themes were found and organized based on the most frequent reports.

## Results

Five themes were identified: 1) sepsis, 2) impaired immune functions: *In vitro *studies, 3) mortality, 4) villous atrophy, 5) duration of dependency on TPN after bowel resection. Table 3 provides the demographic characteristics of the studies included in this systematic review.

**Table 3:** Demographic characteristics of individual clinical trials included in this review

Studies	Participants (Age)	Interventions	Results
Pierro, van Saene, Jones et al. (1998) [18]	94 infants on PN (median age 37 weeks)	94 infants were on TPN. Throat and rectal swabs (surveillance cultures) were obtained before and twice a week after TPN started. Cefotaxime and metronidazole were given for prophylaxis, then blind therapy with a combination of Gentamicin and teicoplanic was given at the onset of sepsis. Blood cultures (central/peripheral) were sent.	41 patients (44%) on PN for 30 days, developed abnormal carriage. Among these carriers, 2 infants developed oropharyngeal E. Coli followed by Klebsiella spp, enterobacter spp, and Pseudomonas aeruginosa. 9 infants had blood cultures positive with enterococci, E. Coli, Klebsiella, Candida, and coagulase (-) staphylococci.
Pierro, van Saene, Donnell et al. (1996) [17]	94 infants, median gestation was 37 weeks	Surveillance cultures of oropharynx and gut were obtained at the start of TPN and thereafter twice a week. Blood cultures (central/peripheral) were sent.	15 infants developed sepsis. 10 patients experienced septicemia. 6 patients had bacterial translocation, and overgrowth of bacteria observed in 9 patients.
Andorsky et al. (2001) [96]	Total of 30 infants with SBS (age <30 days) NEC = 13, Intestinal atresia = 9, Gastroschisis = 5, Malrotation/volvulus = 3	Median residual small bowel length was 61 cm. All infants were on TPN. The shortest duration of PN use was 101 days, and the longest was 3287 days, and median was 245 days.	Of the 30 patients in the study, 20 (67%) were weaned from PN; 9 of the 10 TPN-dependent infants died from infection, cardiac arrest while receiving TPN.
Okada, Klein, van saene et al. (2000) [7]	41 babies enrolled (<4 mos. old). Gastroschisis = 11, (NEC) = 7, intestinal atresia = 3, diaphragmatic hernia = 2, esophageal atresia = 2, infant with no GI problems = 11, control infants = 5, and healthy adult volunteers (control) = 5.	5 infants receiving TPN for more than 10 days. 5 infants on normal enteral diet. Coagulase-negative staphylococci were added to whole blood from control patients receiving TPN.	Body weight was significantly lower in patients receiving TPN. The blood from control group killed 65% of the coag-neg. Staph, while the blood from long term TPN group failed to kill this organism.
Okada, Klein, Pierro et al. (1999) [20]	Surgical infants (NEC, gastroschisis)= 5, infants (control)= 5, healthy adults (control)= 5.	5 surgical infants on long term TPN (>10 days), 5 infants on normal enteral diet, 5 healthy adults.	The percentage of bacteria killed by the neutrophils increased with time. However, the ability of killing was significantly lower in infants on TPN.
Okada, Papp, et al. (1999) [19]	5 enterally fed infants (age<6 mos), and 6 healthy adults	Fasting blood samples: A) 10 ml normal saline (N/S) B) 0.1 ml TPN in 9.9 ml N/S C) 1 ml TPN in 9 ml N/S D) 10 ml TPN	In infants, 1 ml of TPN in 1 ml blood produced a significant decrease in TNF-α production.
Weber (1995) [94]	21 infants and children with short bowel length (<80 cm) on TPN through central line. 20 infants without SBS (13 NEC, 4 atresia, 1 gastroschisis, 2 volvulus) had surgery	No enteral feeding for 7 to 14 days during the post-op period. Blood cultures from central line and peripheral line were sent to identify the organism	6 patients had 8 separate episodes of sepsis before enteral feeding was began. After enteral feeding started, 16 patients had 67 episodes of bacteremia.
Chaet et al. (1994) [5]	32 children with SBS Gastroschisis = 3 Volvulus = 5, NEC = 8, Atresia = 8, Hirschprung's = 5	32 children with residual small bowel length <100 cm (range 14-94, median 40). All patients required TPN support for minimum 2 months. 10 patients were on TPN for>3 years.	4 patients died. Two of these deaths were from complications of TPN, and other 2 had pneumonia and respiratory failure secondary to broncho-pulmonary dysplasia. All four patients were TPN dependent up to the time of their deaths. One of these death patients bowel length was 30 cm and ICV was intact while the other did not (bowel length, 15 cm).
Kaufman et al. (1997). [98]	49 neonates with SBS, NEC = 20, Atresia = 12, Gastroschisis = 9, Volvulus = 8.	Infants with SBS required TPN for more than 3 months after initial surgery. Oral feeding was permitted in small volumes. Patients went home with TPN.	42 patients were able to wean completely from TPN. Bacterial overgrowth was diagnosed in all 7 children who were receiving TPN. Occurrence of bacterial growth was related to small bowel length. 6 of them died.
Sondheimer et al. (1998) [97]	44 infants NEC = 14, Atresia = 6, Gastroschisis = 4 Volvulus = 2, unknown = 10	Almost half of 32 infants had 50% or more of the estimated intestinal length resection. The remaining 12 infants had 10-50% of bowel resection.	Of the 44 patients, four patients have died from liver failure while on TPN. Seven patients depended on TPN from 40 to 129 months. The rest, 27 patients were off TPN after 36 months of age. Outcome of 10 patients unknown.
Bines et al. (1998). [95]	4 patients with SBS (6 months to 5 yrs), NEC = 2, Volvulus = 1 Hirschprung = 1	All patients had central line. Patients received pregestemil formula via continues GT, while on TPN, then study formula (Neocate) was given	All patients were able to discontinued TPN within 15 months of initiating the study formula. After tolerating enteral study formula, morbidity and hospitalization reduced dramatically.
Rossi et al. (1993) [93]	7 children, ages 9 months to 17 years. 3 children with inflammatory bowel disease (IBD) were on TPN for one month. 4 children with SBS on TPN for 7 to 54 months. 22 infants (control) on normal diet.	All patients while on TPN for one month, underwent to intestinal endoscopy and biopsy. Of these patients: 7 on TPN, and had upper intestinal biopsy. 4 patients required TPN for >9 months	Biopsies from patients in the IBD group didn't show atrophy. 3 patients on long-term TPN for SBS had very mild (grade I) villus atrophy.
Dahlstrome (unpublished, 1988) [90]	29 children (4â€“111 month) SBS = 11/9 on TPN/ PPN (small bowel<25 cm) pseudoobstruction = 7 on PPN immunodeficiency = 1 radiation enteritis = 1 28 (control) healthy children (10 USA and 18 Swedish the same age group)	Group I children absorb <5% of their daily caloric intake; Group II children was 30-70%. Home-TPN was given each night, and children were encouraged to eat daytime as much as possible for an average of two years.	After two years of long-term TPN, children had abnormal lymphocyte count, low levels of serum albumin and protein in-group I. Four children developed selenium deficiency, and 15 children on PN for 3 yrs had significantly low Hb and Hct compared to controls. Eleven of 29 children died from low lymphocyte count. Seven died (5 from SBS, 1 from pseudoobstruction, 1 from immune deficiency), 4 from TPN induced cholesistatic liver disease and from bacterial septicemia.

PPP:Partial parenteral nutrition, PN: Parenteral nutrition, Hb: Hemoglobin, Hct: Hematocrit

### Sepsis

Six of the 13 trials reported bacterial overgrowth, sepsis, or septicemia [58,60,90,94,96,98]. In the first study, [96] investigators reported that two children died from gram-positive central venous catheter infections. In the second study [98], researchers compared a total of 49 neonates with 7 SBS who were on TPN and 42 weaned children. It was ascertained that the occurrence of bacterial overgrowth was related to small bowel length. Eleven TPN-weaned subjects who had bacterial overgrowth had a mean bowel length of 54 cm as compared to 105 cm in those without bacterial growth. In Pierro and colleagues' study [58], 41 children whose surveillance cultures developed abnormal carriage. These investigators stated that infants receiving TPN are at a very low risk of developing sepsis and septicemia as long as their surveillance cultures reveal normal flora only. Conversely, the presence of abnormal PPM in the throat and/or the gut increases the risk of sepsis and septicemia to about 25% of the population art risk. They have also stated that the sicker and more physiologically stressed infants would not regain normal gut function as fast as neonates who were doing clinically better.

In the third study [60], the purpose of the investigation was to demonstrate an association between microorganisms that were carried in the digestive tract and present in the blood of infants with sepsis who were receiving TPN. Sepsis occurred in 15 patients (43 episodes) and 10 patients experienced septicemia (24 episodes). Six infants had 15 episodes of bacterial translocation due to *E. coli*, *Klebsiella*, *Candida *species and enterococci. Eight patients had nine episodes of septicemia caused by coagulase-negative staphylococci. The researchers concluded that there was an association between septicemia and elevated serum bilirubin level, indicating TPN related cholestasis.

Dahlstrome [90] investigated a total of 29 children, most of whom had SBS and had been on TPN for 2 years. Children receiving TPN for an average of 2 years developed low biochemistry levels. The children were divided into two groups. The children in group I were estimated to have absorbed less than 5% of their daily caloric intake from the intestinal tract, while intake in the children in group II was 30-70%. Based on previous animal studies, the investigator predicted that the low lymphocyte count was related to extensive bowel resection, (the average bowel length of group I was<25 cm). Eleven of these children eventually died from TPN related sepsis, septicemia, central line infections, or cholestatic liver diseases.

### Impaired immune functions: *in vitro *studies

In three *in-vitro *control studies, investigators elucidated that long-term TPN in infants suppresses specific mechanisms of immune functions [7,51,67]. In the first study, Okada and colleagues (1999) investigated the effects of TPN solution on neutrophil phagocytosis and whole-blood cytokine production in response to coagulase-negative staphylococci in an *in vitro *challenge in five enterally fed infants (age<6 months) and 6 healthy adults. They found that in infants, after 2 hours of incubation with a physiological dose of TPN (1 μ of TPN in 1 ml of blood) there was a significant decrease (*P *< 0.05) in tumor necrosis factor alpha (TNF-α) production.

The other two *in vitro *controlled-clinical trials focused on the cellular mechanism of neutrophil dysfunction, and the whole blood bactericidal activity against coagulase negative staphylococci in the infants receiving TPN [7,67]. These investigators used an *in vitro*-controlled whole blood model to measure the host bactericidal activity against coagulase-negative staphylococci. When researchers added coagulase-negative staphylococci to whole blood drawn from control patients receiving enteral feeding, the median killing of coagulase-negative staphylococci was 65%. In contrast, blood from infants receiving long-term TPN failed to kill this organism as effectively. Infants on long-term TPN had the lowest levels of both whole blood and intracellular killing of coagulase-negative staphylococci due to a defect in neutrophil function. These investigators reported that there was a negative linear correlation between the duration of TPN in days and killing of coagulase-negative staphylococci (P = 0.002). For every additional week of TPN, there was a 7% reduction in killing of coagulase-negative staphylococci. These investigators also noted that there was a significant positive correlation between neutrophil count and killing of coagulase-negative staphylococci (r = 0.80, P = 0.001).

### Mortality

A total of 34 TPN complicated deaths were reported in five of the 13 clinical trials. Of these 34 deaths, 7 children died from SBS, 12 from sepsis, respiratory infections, and low immune deficiency, and other 15 from liver failure [5,90,96-98].

### Duration of dependency on TPN after bowel resection

There were only three studies, retrospective and case study that showed the dependency on TPN in infants who had undergone resection of the small intestine [95-97]. As reported in these studies, there were only 17 children who were totally dependent on TPN, and nine of them died while on TPN. In one of these studies, investigators also noticed that no child successfully discontinued TPN after 36 months of age [97]. They concluded that children dependent on TPN at or more than 36 months of age are permanently dependent on TPN. Andorsky and his co-workers [96] also emphasized that residual bowel length was highly correlated with duration of TPN. Their conclusion was that remaining functional small bowel length was an independent predictor of successful weaning.

Although most children had difficulty being weaned from TPN in other studies, Bines and her co-workers [95] were able to wean all four children from TPN within 15 months of initiation of a study formula. They used an amino acid-based complete infant formula (Neocate, SHS Inc, Rockville, MD, USA) as enteral feeding. Small bowel length of these children was documented between 40 cm to 80 cm with no ileocecal, and in only one child had 13 cm with ileocecal present. All patients had repeated esophagogastroduodenoscopy and colonoscopy or jejunoscopy with findings of mild, nonspecific neutrophilic inflammation involving areas from biopsy specimens from upper and lower intestinal tract.

In addition, two other studies used enteral formulas while children were on TPN. Andorsky and colleagues [96] fed the patients with continuous breast milk or protein hydrolysate formula. Of the 30 patients in the study, 20 were weaned from TPN. Based on their report, median residual bowel length was 61 cm, and ileocecal valve was preserved in 57% of the patients. There was a significant high correlation between use of breast milk and shorter the duration of TPN. Infants who received breast milk were weaned from TPN in 290 days versus 720 days in non-breast-fed infants.

### Villous atrophy

In two separate clinical trials, researchers [93,95] examined whether the effects of TPN on the intestines of children are similar to those reported in animals. In the Rosi and colleagues' study, a total of 32 children (ages 9 months to 19 years) were involved. Based on the biopsy results, the investigators reported that 3 of the 4 patients receiving long-term parenteral nutrition for short-bowel syndrome (TPN >7 months) had mild villous atrophy. Biopsies from 3 patients in the inflammatory bowel disease (IBD) group (TPN = 1 month) did not exhibit atrophy. Bines and colleagues [95] also reported similar results; in their study only one of four children (bowel length, 40 cm) had partial villous atrophy of the small intestinal mucosa.

## Discussion

Systematic reviews focus on empirical studies and seek to summarize past research by drawing overall conclusions from many separate investigations that address related or identical hypotheses [101]. The purpose of this descriptive systematic review was to assess the literature documenting whether TPN negatively influences gut barrier function and is associated with increases in intestinal bacterial overgrowth, bacterial translocation, increases in mucosal permeability, decreases in S-IgA levels, and changes in mucosal architecture in infants and children after small bowel resection. Thirteen clinical trials in children from 1990 to 2001 examined the effects of TPN on infection, mortality, impaired intracellular immune functions, villous atrophy, and long-term TPN dependency. These studies were conducted in four countries from various disciplines; they ranged in size from 4 to 94 children, and age from approximately 4 months to 17 years old, with the majority of children spending time in hospital.

Numerous experimental studies suggest that long-term TPN has harmful effect. Evidence in experimental studies about TPN's effects on mucosal immunity and villous atrophy is very convincing [9,14]; however, findings in human subjects were inconsistent. Although animal models provide us wealth of evidence and continue to offer valuable information to apply to clinical conditions, Rossi and coworkers [93] argue that extrapolating these results to apply to the human model is inappropriate. Their study results showed that infants with SBS on TPN for more than 9 months had only minimal grade villi atrophy. They concluded that humans are more resistant to hypoplastic intestinal changes induced by TPN; and effects in humans seem to require longer periods of TPN. In adults, 203 surgical patients who had at least 10 days of preoperative TPN without enteral nutrients, no significant decrease in villous height or increase in bacterial translocation were noted compared with those on enterally fed controls [102]. Guedon and colleagues [103] performed biopsies in the duodenum of seven adults (all with inflammatory bowel disease) before TPN, after about 3 weeks of TPN, and after discontinuing TPN and restarting oral feedings. They noted no change in gross villous morphology with only moderate decrease in microvillus height after 21 days of TPN. Buchman and colleagues [3], however, found a significant decrease in villous height after 2 weeks of TPN study involving eight healthy volunteers. Although mucosal thickness decreased significantly, in contrary to animal studies, villous architecture was preserved after TPN; and five days of enteral refeeding was sufficient to reverse the intestinal morphologic changes. Pironi and colleagues [104] performed endoscopic biopsies in 2 adult patients who underwent long-term TPN for the treatment of a postoperative enterocuteneous fistula, and their results showed changes in villous height and crypth depth after 2 and 3 months of TPN; two months after oral refeeding, the values of morphometric parameters were significantly greater than those observed after TPN and were similar to those controls. Groos and colleagues [105] also conducted a study involving 20 adults to investigate whether TPN causes morphological changes in intestinal mucosa of human adults similar to those observed in animals experiments. They found jejunal mucosal atrophy, and a remodeling of luminal surface architecture with disappearance of cell shedding.

### Bacterial translocation

In this review, bacterial translocation was reported only in one study [60]; it stated that 10 children experienced 24 episodes of septicemia, but only 6 out of 94 infants had bacterial translocation. Andorsky [96] reported that 9 of 10 children died from infection while on TPN, but no statement has been found whether those children died from infection or from other causes. In a large review, Lipman [106] critically assessed the effects of TPN and enteral nutrition, and did not find any convincing evidence that TPN promotes bacterial translocation or enteral nutrition prevents bacterial translocation in humans.

In a large critical review, Lipman [107] examined the existence of bacterial translocation and the effects of enteral nutrition on bacterial location using both animal and clinical trials. He did not find any evidence supporting that enteral nutrition preserves gut barrier functions and prevents bacterial translocation. He concluded that bacterial translocation is an independent of intestinal structure; and also the villous atrophy seen may be species specific.

Pierro and colleagues [58] used throat and rectal swabs as surveillance culture samples twice a week to investigate whether carriage of abnormal flora was associated with increased risk of sepsis and septicemia in children receiving TPN; and they concluded that infants receiving TPN are at very low risk of developing sepsis or septicemia as long as their surveillance cultures reveal normal flora only. However, the presence of abnormal PPM in the throat or the gut increases the risk of sepsis and septicemia by 25 % [58]. Surveillance samples are defined as specimens obtained from body sites were PPM are normally carried by the digestive tract.

### IgA

The importance of enteral stimulation on the mucosa-associated lymphoid tissue (MALT) system was studied in neonates who died soon after birth. Histological examination of the biopsies showed that infants who received enteral stimulation showed clear evidence of B cells and T cells within the mucosa; whereas, parenterally fed infants who died had a villous atrophy. Gut and bronchus samples were obtained and related to time of death of infants who died of sudden infant death syndrome between two weeks and 90 moths after birth. IgA plasma cells first appeared in the gut and later in the bronchi as the system matured. Plasma cells increased rapidly over time as IgA plasma cells predominated by three weeks in the gut and six weeks within the bronchi [108].

In this review, none of these studies have tested whether TPN decreases S-IgA levels in intestinal mucosa causing increased bacterial translocation. Although there was only one study focused on the changes in IgA production during TPN in 8 healthy human volunteers [109], the results of this study did not show significant differences in the number of immunoglobulin-containing cells. Intestinal immune function was not affected by 2 weeks of TPN. Woodcock and colleagues [61] also investigated whether bacterial translocation is associated with changes in gut immune function in 22 adult patients (11 of whom were positive bacterial translocation and 11 negative). However, these patients were not on TPN. The study results showed a significant increase in immune functions, especially higher numbers of plasma cells and IgA and IgM values in small bowel mucosa of patients in whom bacterial translocation has been positive.

### Hyperglycemia

In this review *in vitro *studies on infants and children demonstrate that TPN inhibits functions of neutrophils, cytokines, and bactericidal activity of phagocytosis [7,51,67]. Investigators found that cytokine production after bacterial challenge was directly impaired by addition of TPN solution. In these patients calorie intake varied from 100 to 130 kcal/kg/day; and TPN emulsion consisted of carbohydrate 15 to 18 g/kg/day, amino acids 2-3 g/kg/day, and fat 3 to 4 g/kg/day. Although these studies do not state whether these children's serum glucose levels were high, perhaps TPN-induced hyperglycemia may have also contributed to dysfunction of neutrophils. It has been hypothesized that the elevation of blood glucose in patients receiving TPN may be associated with complications, such as immunosuppression [110]. Glucose concentrations above 220 mg/dL have been shown to glycosylated immunoglobulins, causing a significant reduction in opsonic activity, which adversely affects wound healing and immunity [111].

There is a large body of evidence demonstrates that there is a positive correlation between high serum glucose levels and increased infection rates in acutely ill patients in critical care medicine. Insulin therapy seems to be beneficial in sepsis patients [54]. Rassias and colleagues recently performed two comparative randomized control clinical trials one involving 30 non-diabetic adult cardiac patients (15 in each group) [112], and the second clinical trial involving 26 diabetic adult cardiac patients (13 in each group) [113] who were scheduled for elective cardiac surgery with cardiopulmonary bypass surgery. The experimental group received intensive insulin treatment while the control group received standard insulin therapy. They found a significant increased in neutrophil count and neutrophil functions in intensive insulin therapy group. They suggest that hyperglycemia leads to increased endothelial adherence of neutrophils and may lead to lower white blood cells in hyperglycemic patients.

Since its introduction, TPN has been a life saving nutrient to severely malnourished patients. It was believed that "if some nutrition is good, more is better" [114]. Even in treating diabetic patients in the hospital, for decades, an oral tradition was passed down from clinician to clinician, "keep the patient a little sweet" [115]. It has been recently recognized that in the presence of sepsis an increased intake of energy, or overfeeding the patients with carbohydrates or fats, increases the risk of complications, which contributes to hyperglycemia and sepsis. This TPN-induced and/or physiological stress-induced hyperglycemia may have also contributed to increased rates of sepsis [39,114]. In a careful analysis of stress-induced hyperglycemia in 102 non-diabetic patients receiving TPN, subjects who received dextrose at >5 mg/kg/min, had 50% chance of developing hyperglycemia [116]. In contrast, dextrose infusion at < or = 4 mg/kg/min, risk was substantially reduced. Therefore, to prevent hyperglycemia and infection complications in hospitalized patients, TPN dextrose infusion rates should be at < or = 4 mg/kg/min [117].

Two randomized prospective clinical trials [118,119] of aggressive insulin therapy now have revolutionized our current philosophy about treating critically ill hospitalized patients [53]. In the first study, a total of 620 patients (306 patients randomized to treatment with insulin-glucose infusion followed by multidose subcutaneous insulin for ≥ 3 months and 314 to conventional therapy) [119]. In the second study, Van den Berghe and coworkers [118] conducted a large prospective, nonblinded, randomized clinical trial (a total of 1548 surgical patients in ICU) of intensive glycemic control (glycemic goal of 80-110 mg/mL [4.4-6.1 mmol/L] compared with conventional treatment (maintenance of blood glucose at a level between 180 and 200 mg/dL). At the end of the study, results showed that intensive insulin treatment reduced episode of septicemia by 46%, and overall mortality rate by 32% during their stay in ICU.

Hyperglycemia is commonly seen in stressed patients during administration of TPN or other glucose-containing solutions. Stress may also induce insulin resistance in adipose tissue, liver and heart [120]. However, the treatment of hyperglycemia starts only after glucose levels have exceed 200 to 250 mg/dL (11â€“14 mmol/L) because it was believed that avoidance of hypoglycemia and its potential consequences is more important than glycemic control while patients are hospitalized. The most recent target range for plasma glucose in the hospital are: preprandial = <110 mg/dl; peak postprandial = <180 mg/dl; and for critically ill patients = 80-110 mg/dl [53].

Some studies reported that the complications during TPN were associated with the residual or the remaining bowel length in patients with SBS. The longer the residual small bowel, the shorter the duration of TPN, and the fewer infections [5,96]. The results of Dahlstrome's [90] study revealed that children whose bowel length was <25 cm developed very low levels of circulating lymphocytes causing immuno-competence due to very short bowel length, and not ingesting the food antigens which are the main stimulants of lymphocytic proliferation and immunoglobulin (IgG, IgA) synthesis. Dahlstrome predicted that lack of ingested antigens might have caused the low circulating lymphoctes, which resulted in infection. He found that enteral support in combination with TPN did improve, but not normalize the plasma amino acid concentrations in the children investigated.

### Enteral versus parenteral nutrition

It has been suggested that enteral stimulation is a required component to protect gastrointestinal and respiratory immunity via increased levels of mucosal IgA [15,121]. Enteral nutrition refers to nutrition either ingested orally or delivered to the stomach or intestine by tube via nasogastric (NG) tube, gastrostomy tube (GT), nasojejunal (NJ) tube, or jejunostomy tube (JT). Evidence showed that enteral nutrition is less expensive, but not safer, or more physiologic than TPN [106]. In a prospective observational nursing study [122], 64 elderly patients who were fed by an NG tube in an internal medicine. The type of formula was not stated in the study, but based on this report; most patients had electrolyte imbalances, tube dislodgements, hyperglycemia, diarrhea, pulmonary aspirations, and nasal ulcers. In another prospective observational study [123], a total of 153 critically ill patients also were fed with NG tube. Before each feeding, gastric residual volume was checked by using 50-ml syringe by aspirating the tube. The study results showed that there was a negative relationship between high gastric residual volume and days in the hospital stay. High gastric aspirate volume was associated with a higher incidence of nasocomial pneumonia, a longer length of hospital stay, and higher mortality.

### Effects of early enteral feeding on bacterial translocation

In Weber's study [94], 21 infants and children with SBS were begun with enteral feedings via continuous drip technique with an elemental formula (Pregestemil:Mead-Johnson, Evansville, IN), after 3 weeks of postoperative period. Results showed that after enteral feeding began, 76 % of children had 67 episodes of bacteremia. The investigator suggests that early enteral feeding increases infection. However, Marik and Zaloga [124] performed a systematic review of 15 PRCT included 603 patients to evaluate the effect of early enteral nutrition on outcome of critically ill and injured patients. The results of this study showed that there was a significantly lower risk of infection in 19 % of the patients who received early enteral diet compared with 41 % in the delayed group. This study concluded that early feeding decreases infectious complications and length of hospital stay. In a large review, by pooling data from four PRCT involving 142 patients who had gastrointestinal cancer, Klein et al. [125] compared early postoperative JT feeding with the usual advancement of oral diet as tolerated. Based on the aggregated data, there were no significant differences in postoperative morbidity or mortality.

However, Daly et al. [126] performed a PRCT involving 85 adult the patients (41 on supplemented diet, 44 on standard diet) with cancer of upper gastrointestinal tracts. The patients who were fed early postoperatively [on the first postoperative day] via JT with a formula supplemented with arginine, ribonucleic acids, and ω-3 fatty acids had fever infections, minimal wound complications, and a shorter duration of hospitalization compared to those who received standard formula. Braga at al. [127] also performed a PRCT involving 166 patients undergoing gastrointestinal surgery to evaluate the impact of the route of administration of artificial diet, IMPACT^Â® ^(Novartis Nutrition, Bern, Switzerland) supplemented with arginine, ribonucleic acid, and omega-3 fatty acids versus standard diet on outcome. Their results showed that early postoperative enteral infusion of nutrients is safe and well tolerated and stimulates an early return of bowel function.

### Immunonutrition

There has been a considerable amount of interest in recent years in the use of alternative specific gut substrates. The term *immunonutrition *has been coined to describe molecular compound that, while being dietary components, such as glutamine, arginine, ω-3 fatty acids, ribonucleic acid, also influence immunologic response mechanisms when added to standard TPN solutions or enteral nutrition [128-130]. Glutamine, a nonessential amino acid, is considered to be the principal respiratory fuel for enterocytes, colonocytes, lymphocytes, and macrophages, and is a precursor for nucleotides synthesis and for glutathione, an important antioxidant that may be protective in a variety of circumstances. It has been hypothesized that during the stress, glutamine becomes conditionally essential amino acid.

In the late 1980s, glutamine has been the most extensively studied substrate in animal research, especially in rat TPN model. It has been suggested that parenteral or enteral glutamine supplements could dramatically effect on the maintenance of GALT; and glutamine-enriched TPN significantly attenuates the mucosal hypoplasia associated with prolonged TPN following massive bowel resection [131-134]. Therefore, it was hypothesized that glutamine supplementation in human modal would have similar effects [77]. Encouraged by findings in animal investigations, in 1991 Darmaun and colleagues [135] first time reported a reduced rate of glutamine turnover in short bowel syndrome [adult] patients, and in 1994 in pediatric patients [136]. They suggested that the small intestine was a target organ for glutamine utilization in both adults and children. C.K. Ogle (unpublished, cited in Alexander, 1993) [137] also has shown that excess glutamine added to culture media can improve antimicrobial killing by neutrophils isolated from blood of burn patients and normal controls. Alexander also argues that infection can be reduced 75% and hospital stay by 20% by immune enhancement via enteral nutrition.

In the following years, many controlled trials and case series were conducted to demonstrate the benefits of immune enhancing substrates to the intestinal adaptation process in patients with SBS during long-term TPN [138-147]. The results of these published studies have been conflicting. Although glutamine supplementation has been shown to be value in pediatric patients [38,148], Duggan and colleagues [149] conducted a randomized double-blinded, controlled clinical trial of enteral glutamine supplementation in 20 infants with SBS. At the end of study, there was no significant difference in weight and length gain between two groups. Two of nine infants in the glutamine group developed urinary and bloodstream infections. Overall, this study results showed that glutamine supplementation was safe, but had no effect on duration of TPN, tolerance of enteral feeds, or intestinal absorption or barrier function. However, in a PRCT pilot study Barbosa and colleagues [150] examined the tolerance and efficiency of glutamine in a pediatric intensive care unit setting with nine mechanically ventilated infants (treatment group = 5, and control group = 4). Their results showed that tolerance to glutamine-supplemented diet was good. Bacterial infections reported in 75% of the placebo population and 20% of the treated infants. There were two deaths from microbial infection in control group, suggesting a beneficial effect of glutamine.

However, Scolapio and colleagues [142,151] conducted a series of double-blind, 6-week, placebo-controlled, crossover trial in 8 adult patients with SBS to assess: 1) the effects of growth hormone, glutamine, and high-carbohydrate-low-fat (HCLF) diet on body composition, and 2) to evaluate the potential mechanisms that might explain the beneficial effects of immunonutrition treatment. Active treatment consisted of subcutaneous recombinant human growth hormone and oral L-glutamine. In this study, two patients developed carpal tunnel syndrome that resolved 5 days after discontinuation of active treatment. Two patients noted sleep disturbances while on active treatment. Patients developed peripheral leg edema and weight gain; this combination therapy resulted in a significant increase in sodium and potassium absorption. Two studies [145,152] also reported similar findings in addition to severe hand pain, and gynaecomastia in a male patient. Two systematic reviews [138,153], and two large critical reviews [77,125] conclude that glutamine and growth hormone at present time can not be recommended in short bowel syndrome; more large-scale studies, or multicentered clinical trials are required.

Currently, two commercially prepared immune-enhancing enteral formulas are available in the market, IMPACT^® ^(Novartis Nutrition, Bern, Switzerland) containing glutamine, arginine, dietary nucleotides and fish oil; and Immune-Aid (McGaw, Irvine, CA) containing glutamine, arginine, nucleic acids, and omega-3 fatty acids. Some studies indicate that these immune-enhancing diets demonstrated successful results or benefits in studies of sepsis, immunity, altered intestinal permeability, inflammation, including reduced mortality and the length of hospital stay [154-156].

Some studies also show that the use of breast milk in SBS contains high levels of immunoglobulin A (IgA), nucleotides, leukocytes, and other components that bolster the neonate's immune system[20]. In a study, a continuous infusion of small amounts of breast milk in infants with SBS is also resulted in a shorter duration of TPN compared to control group [96].

Overall, findings are inconclusive. Immunonutrition may be species or patient specific.

## Implications for evidence-based nursing practice

### Multidisciplinary nutritional team

The successful management of infants and children with SBS is demanding and requires a multidisciplinary approach. The care of patients with SBS can become so complex that many patients with SBS almost lose their primary physician and become patients of multiple specialists. Long-term care is, therefore, best delivered by a variety of healthcare professionals, including the gastroenterologists, surgeon, nurses, nutritional support team, care coordinators, microbiologist or infection control team, clinical chemist, pharmacists, social workers, and psychologist with expertise in infant feeding difficulties. Patients cared for in this way have better outcomes [25,148,157].

Infants go through a critical developmental phase in feeding at about 6â€“12 months, and those who do not receive oral feeds at that time will loose the window of opportunities for sucking and swallowing coordination, and will have long-term eating difficulties [21,158]. In addition, children fed enterally or parenterally may have forgotten or never learned the association of with hunger, oral feeding, and satiety; they develop "defensive oral behaviors," such as gagging, choking, and vomiting. For a sick child, if this important developmental phase is ignored during the first year of life, flavor, texture, odor, and extreme temperature can be overwhelming. It is important that the pediatric nurses should consult pediatric occupational therapist and speech therapist, and behavioral psychologist to work with these children [159].

### Clinical management

The management of neonates and children with SBS continues to provide a major challenge for practitioners [160]. A group of pediatric surgeons critiqued the current clinical management of SBS in children, which is mostly based on "trial and error" regimen. They strongly recommended that prospective, randomized controlled trials should be established, and an evidence-based rather than a "gut-feeling" -based approach to be used in SBS children, such as intestinal permeability and sugar absorption tests, evaluation of adaptation by gut hormone production, immunohistochemistry, and other new techniques which are still in experimental stage [154]. Pediatric nurses should, therefore, provide an evidenced-based nursing care to these children with the support of APRN.

Hypersecretion is seen in infants and children during the initial first 6 months after surgery and a start of enteral feeding. The H_2 _blockers and proton pump inhibitors are effective for reducing gastric fluid secretion, and therefore, will also reduce fluid losses during this period [77,161].

The diagnosis of bacterial overgrowth is determined by aspiration of the jejunum and demonstration of increased bacterial contents by culture. Since this procedure is invasive and infeasible in some sick infants, breath hydrogen test can be used easily as noninvasive diagnostic test [162]. The breath hydrogen test is an oral test that uses the measurement of hydrogen in the breath to evaluate carbohydrate malabsorption and bacterial overgrowth in small intestine. Hydrogen gas is produced by bacterial fermentation of undigested carbohydrate that reaches the colon, enters the portal and systemic venous return, and is then released in the breath. After fasting 4-6 hours, the child ingests a load of carbohydrate (12 g/kg, maximum 50 g), and the end-expired air is collected in sealed plastic bags by aspirating 5 mL of air after each breath to total of 20-30 mL via nasal prong attached to a face mask at timed intervals up to 2 hr after ingestion. Malabsorption of any carbohydrate can be evaluated. The child should not be taking antibiotics at the time of the study because these drugs alter the colon flora and suppress hydrogen gas production [163-165]. In one of the clinical studies, investigators used 50 mg ^13^C-xylose in children ages 3 to 12 years for breath hydrogen test [166]. ^13^C-xylose is a safe, nonradiactive isotope that has recently been developed (Martek, Columbia, MD). This study results showed that all patients with bacterial overgrowth had positive breath test results (100% sensitivity).

For infants prone to overgrowth, routine scheduled antibiotic treatment may be useful. D-Lactic acidosis has been reported in children with bacterial overgrowth, causing metabolic acidosis, drowsiness, and confusion. This diagnosis should be considered in a child with SBS who presents with metabolic acidosis, high serum anion gap, normal lactate level, and without urinary ketones [161]. Treatment for this syndrome is to start oral metronidazole, neomycin, vancomycin, and avoidance of "refined" carbohydrates [77].

### Dietary management

Optimal timing of the initiation of enteral nutrition has not been established [161]. Patients who undergone massive bowel resection require TPN for the first 7-10 days. American Gastroenterological Association (AGA), based on the current literature review, suggests that nutritional therapy should not be introduced until the patient is hemodynamically stable and fluid management issues are relatively stable.

Although the optimal formula for feeding infants and children with SBS is not well established, elemental amino acid based hydrolyzed formulas are suggested by some clinicians and have been shown to be well tolerated [95]. Dietary protein is first digested, and then absorbed as dipeptides and tripeptides. Therefore, it was reasoned that dietary protein provided in a predigested form would be more readily absorbed [77].

A prospective, randomized, cross-over trial compared two protein hydrolysate formulas given by continuous nasogastric infusion to six malnourished infants with SBS aged 1-13 months [167]. Although there was good tolerance for both formulas and satisfactory weight gain, and also energy absorption was the same, but differences in the amount of malabsorbed carbohydrate existed. These researchers suggested that protein hydrolysate formulas should be reformulated with a lower concentration of carbohydrates and a higher one of fat.

A controlled trial compared two feeding regimens, continuous intragastric feedings and intermittent oral feeding, in nine infants with protracted diarrhea and malnutrition and two infants with surgically created short bowel [168]. Continuous nasogastric feeding caused significant increases in enteral balance of the major nutrients, whereas intermittent feedings resulted in negative or only slightly positive enteral balance. There was also a significant increase in body weight during the continuous feeding as compared to the intermittent feeding. The authors suggest that improved enteral balance can be achieved with continuous feeding in infants with short bowel disease [168].

In infants under 1 year of age with SBS who might have dilated gut, poor motility, or bacterial overgrowth, increased epithelial permeability to food antigens occurs frequently and may result in the development of allergic reaction to any protein in the formula. In order to reduce the risk of allergic injury to the gut, a hypoallergenic formula, such as Nutramigen, Pregestemil(Mead Johnson Laboratories, Evansville, IN), and Alimentum (Ross laboratories, Columbus, OH), should be used during the first year of life. To further decrease the risk of allergic reaction in highly susceptible infants, an amino acid based formula should be used. Neocate (SHS Inc, Rockville, MD, USA) is the only one listed in this category for infants. Elecare (Ross Laboratories), which is quite similar to Neocate, was formulated for use in children over 1 year of age [169].

Initially slow introduction of continuous enteral feeding via a nasogastric or gastrostomy tube feeding is beneficial to reduce emesis, diarrhea, and maximally saturate carrier proteins. Rationales for this that constant saturation of mucosal digestive enzymes and transport carriers should be optimize absorption. Regardless of the length of small intestine, some oral, even if only 1 or 2 ml per day, and a few ml in continuous fashion should be offered from an early stage [158].

A diluted infusion is initiated continuously at low volumes and increased to 0.67 cal/mL for infants less than 1 year of age or to 1.0 cal/mL in older children. Once the final concentration is achieved in low volumes, enteral feeding rates are advanced, and TPN rates are decreased isocalorically every 1 to 3 days as tolerated [170]. A marked increase in stool loss by 50 % is usually contraindication to advancing enteral feedings. There is no agreement on the amount of stool output that should be accepted. Limits should be imposed on feeding once the stool output exceeds 45 ml/kg per day [161]. However, a higher volume of stool output may be acceptable by some clinicians [171]. If stool losses greater than 45 ml/kg/day or ostomy output strongly positive for reducing substances suggest that enteral feeding advancement should be slowed. Careful fluid and electrolyte management is essential as dehydration can occur rapidly [20,158,161,170].

### Monitoring enteral feedings and verifying tube location

Monitoring patients on enteral feeding is required routine assessment of gastrointestinal, metabolic, mechanical, and growth parameters [172]. Tolerance of enteral feeding is assessed by noting the presence or absence of vomiting, retching, abdominal distention, and diarrhea. For patients receiving gastric feedings, checking for residual formula for every 4 hrs is required to evaluate feeding tolerance or delayed gastric emptying [173]. During continuous feeding, if a single high gastric residual volume is greater than 1.5 times the hourly feeding of formula infusion, this indicates a sign of intolerance. Feeding should be stopped, and clinician should be notified. Gastric residual should be rechecked every 1-2 hours until the residual drops below the greater than or equal to 50% of the hourly rate mark [174]. Decreasing the rate by half for a few hours, rechecking residuals, and slowly advancing is recommended to achieve feeding tolerance. Changing the tube feeding might be beneficial. If this is unsuccessful, child can be placed in the right lateral decubitus position and i.v. metaclopromide (Reglan) can be started to enhance gastric emptying [172,175].

A literature review conducted by Metheny and colleagues [176] found that there was a confusion as how gastric residuals should be handled. Fifty percent of the respondents (registered nurses) from a survey (Mateo, 1996, cited in Metheny et al., 2004) [176] reported discarding the gastric contents and 49% reported re-administering them. Only one study was located that dealt with outcomes of discarding or returning gastric residuals to the patients (Brooker et al., 2000, cited in Metheny et al., 2004) [176]. In this study 35 subjects receiving enteral feedings were randomized to either a discard group or a return group. Repeated measures analysis of variance found no significant differences between the two groups in the body weight, serum electrolyte levels, tube clogging, nausea, and the feeding delays. In the absence of more convincing evidence, Metheny and colleagues [176] suggest for returning gastric residuals less than 500 mL to the patients.

Ensuring correct placement of the feeding tube is also necessary. Although auscultation is the most common method of confirming placement, it has been found unreliable. The best evidence for confirming correct tube placement is X-ray [177]. Metheny and colleagues [177,178] provide evidence-based nonradiographic methods to check the tube location. There are two methods currently available at bedside to nurses to test tube placement during continuous feedings include (1) observing the appearance of fluid aspirated from the tube, and (2) measuring the fluid's pH. Gastric pH usually falls in the range of 1 to 5, whereas intestinal or respiratory pH is usually 7 or higher. If a pH ≤ 6 is a significant indicator of the gastric placement, whereas a pH >6 is an indicator of intestinal placement. Metheny and Stewart [178] also provide additional methods for checking the position of the tube, such as capnography, spring-gauge pressure manometer, enzyme tests (pepsin and trypsin), and bilirubin analysis; however, the most reliable method of tube placement assessment is the radiograph [172].

### Monitoring TPN complications

According to American Gastroenterological Association (AGA) [77], and American society for parenteral and enteral nutrition (ASPEN) guidelines [161], TPN should be infused via single lumen catheter with its tip positioned in either the superior vena cava or inferior vena cava to decrease the risk of infection and trombosis. Tunnelled catheters, implantable port, or percutaneously inserted central catheters (PICCs) should be used at home. Peripheral infusion of parenteral formulations is limited to dextrose concentrations of less than 12.5% [161]. ASPEN suggests that initially, TPN should be started with low dextrose infusion rate [161]. Starting rate is usually half of the hourly rate for the first hour, and then rate is increased to full rate after checking the blood sugar. Blood glucose should be monitored at least 4 times a day, and should be <180-200 mg/dl [77]. Glucose intolerance is the major adverse effect seen during the initial infusion period [179]. Urine sugar and acetone or ketone levels should be less than 2+. ASPEN suggests that dextrose infusion rates in infants and neonates should be 10 to 14 mg/kg per minutes[161]. Continuous insulin infusion (0.01-0.1 unit/kg per hour) has been shown to be safe and effective in managing hyperglycaemia in the neonate. Glucose concentration should be monitored at least every 2 hours, aiming for blood glucose concentration between 100 and 150 mg/dL. In children less than 2 years of age, hypoglycaemia develops rapidly if feedings are delayed or interrupted. ASPEN recommends that TPN be tapered over 1 to 2 hours in infants before discontinuation to avoid hypoglycaemia [161]. Some iv medications need to be administered over 30 to 60 minutes. If the patient has only one line which TPN is running, the nurse should communicate with the paediatric pharmacist to reconstitute the drug with 5% or 10% dextrose if that drug is incompatible with TPN. This will also prevent hypoglycaemia if TPN is temporarily interrupted for iv medication.

Four major categories of complications exist: (1) mechanical or technical; (2) infectious; (3) metabolic; and (4) nutritional [161]. Mechanical or technical complications are usually related to catheter placement, such as pneumothorax, hemothorax, cardiac tamponade, or equipment malfunction. Catheter thrombosis is a significant problem of all central lines. A potential early sign of catheter thrombosis is progressively sluggish or absent blood return on catheter aspiration. Thrombolytic agents are effectively used to dissolve thrombi.

Catheter related infections are the most common complications associated with central line TPN [161]. Localized infections are, such as erythema, tenderness, induration, or purulence that occurs at the exit-site or along the tunnel. Pocket infections occur only with implantable ports. Systemic infections, formerly called catheter sepsis or bacteraemia, are defined as positive culture of the catheter tip or a positive pathogen isolated from both blood drawn through the catheter and peripherally. *Staphylococcus epidermidis*, *Staphylococcus aureus*, and other skin flora are the most common pathogens isolated in patients with systemic infections. Enterococcus and enteric flora are the next most frequently isolated organisms. Catheter-related infections are treated based on the type of infection and pathogen [161].

## Conclusion

TPN is a life saving alternative nutritional support to severely malnourished surgical patients. Its use is indicated to prevent the adverse effects of malnutrition in patients who are unable to tolerate nutrients by oral or enteral routes. Evidence from a large systematic reviews involving 82 randomized control clinical trials on both adult and paediatric population showed that: 1) TPN did not have a significant impact on survival, 2) TPN did not affect either the total or infectious complication rates, 3) TPN had no effect on the duration of hospitalization of surgical patients [180]. Based on the current evidence, TPN seems to be safe and a life saving solution.

## List of abbreviations used

Short bowel syndrome (SBS)

Total parenteral nutrition (TPN).

Immunoglobulin A (IgA)

Secretory immunoglobulin A (S-IgA),

Gut-associated lymphoid tissue (GALT)

Gastrointestinal (GI)

Mucosa-associated lymphoid tissue (MALT)

Necrotizing enterocolitis (NEC)

Migrating motor complex (MMC),

Histamine2 (H_2_)

Nitric oxide (NO),

Nitric oxide synthase (NOS

Nonsteroidal anti-inflammatory drugs (NSAIDs)

Tumor necrosis factor (TNF)

Interleukin-1 (IL-1)

Interleukin-6 (IL-6)

Adrenocorticotropic hormone (ACTH)

Antidiuretic hormone (ADH),

Free fatty acids (FFA),

Pathogenic microorganism (PPM)

Neutrophil polymorhonuclear (PMN)

Prospective randomized clinical trials (PRCT)

Intensive Care Unit (ICU)

Nasogastric (NG)

Gastrostomy tube (GT)

Nasojejunal (NJ)

Jejunostomy tube (JT)

American Gastroenterological Association (AGA)

American society for parenteral and enteral nutrition (ASPEN)

Percutaneously inserted central catheters (PICCs)

## Competing interests

The author(s) declare that they have no competing interests.

## Pre-publication history

The pre-publication history for this paper can be accessed here:


